# A Hospital for Atlanta

**Published:** 1889-04

**Authors:** 


### A HOSPITAL FOR ATLANTA.

From present indications, it appears that Atlanta is at last in a fair way to have a City Hospital. So urgent has become the need of such an institution, that in spite of the inexplicable apathy upon the subject which has so long held possession of the profession most interested in its establishment, and in spite of the self-seeking opposition on the part of some members of that profession, the City Fathers have at length taken hold of the matter in earnest, and have made a beginning by appropriating the sum of ten thousand dollars to be paid during the present year, and recommending the appropriation of a similar sum for each of the two following years. A condition of this appropriation is, that the old Benevolent Home property on Waverly Place, which is now lying idle and useless, shall be converted into cash and added to the Hospital fund, or utilized in some other way in furtherance of the project. The only obstacle in the way of the accomplishment of this plan is the question of the title to the Benevolent Home, which is claimed both by the Association of ladies, through whose efforts it was originally founded and for a long time maintained, and by the Board of Trustees in whose hands the control of the property was subsequently placed. This question is now in litigation, and has never been legally settled.

In view of the charitable purpose contemplated in the raising of the money by which the Home was originally purchased, and in view of the new aspect of the matter developed by the recent action of the City Council, it would âppear to a disinterested observer that both parties to this suit might "pool their issues," and reach an amicable adjustment of the question upon the basis of a community of interests. By acceding to the proposal of the City Council, the original charitable end in view would be much more fully served than in allowing the property to lie idle and useless, as at present, and to be finally eaten up by attorney's fees and costs of court.

It cannot be doubted that if a Hospital can once be built, its support will be ready and abundant. Assurance has already been received from a number of corporations and private individuals that they will contribute liberally to this end. There is a large class of paying patients from outside the city who visit Atlanta for medical and surgical treatment, and who would prefer the pay wards of a well-conducted hospital to the illy-adapted accommodations of a hotel or boarding-house. From this source a considerable revenue might confidently be expected. The various railroads centering in this city, and the various large manufactories which now employ surgeons to attend their injured employès, would certainly find it preferable to support one or more beds in the Hospital for their own exclusive use. Thus only a comparatively small portion of the cost of maintenance would fall upon the city, and this amount would probably be less than that now expended in caring for the sick poor.

The time seems ripe for the carrying of this project through to completion. The medical profession is now practically unanimous in its recognition of the need of a Hospital, the public sentiment is aroused, the municipal authorities have shown, in a substantial way, that they favor the project, and all that remains is for the friends of the enterprise to combine their efforts and transform the wishes and hopes and sentiments of these various parties into tangible results. If this is to be done at all, it must be done at once. Nothing is so dead as a dead boom. If the present Hospital boom is allowed to expire, no efforts of its friends will be able to galvanize the corpse into even a semblance of life. Let the good work go on.

